# Clinical Holistic Medicine: Treatment of Physical Health Problems Without a Known Cause, Exemplified by Hypertension and Tinnitus

**DOI:** 10.1100/tsw.2004.129

**Published:** 2004-08-24

**Authors:** Søren Ventegodt, Mohammed Morad, Isack Kandel, Joav Merrick

**Affiliations:** ^2^ The Quality of Life Research Center, Teglgåstraede 4-8, DK-1452 Copenhagen K, Denmark; ^2^ Division of Community Health, Ben Gurion University, Beer-Sheva, Israel; ^3^ Faculty of Social Science, Department of Behavioral Sciences, Academic College of Judea and Samaria, Ariel, Israel; ^4^ National Institute of Child Health and Human Development, Office of the Medical Director, Division for Mental Retardation, Ministry of Social Affairs, Jerusalem and Zusman Child Development Center, Division of Pediatrics and Community Health, Ben Gurion University, Beer-Sheva, Israel

**Keywords:** quality of life, QOL, philosophy, human development, holistic medicine, public health, holistic health, holistic process theory, life mission theory, group therapy, Denmark

## Abstract

In the medical clinic, we often face health problems that have no known cause, even after a thorough examination. Biomedicine is often unable to find a cure in these situations, leaving the problem unsolved or leaving the patient on a palliative pharmaceutical cure, which is often for a lifetime. In this case, consciousness-based, holistic medicine could be an alternative. Using the theories and tools of holistic medicine wisely, the physician can often provide treatment for the patient. The toolbox of holistic medicine makes it possible to work on everybody because there is always something related to the patients quality of life that can be improved: his love, his purpose of life, and the way he uses his talents, his mind, his feelings, his body, and his sexuality. For treatment in holistic medicine, it really does not matter as much that you cannot give the patient a precise medical diagnosis, because you can always work on the patient with the intention of healing his or her whole life and existence. It is quite a paradox that many of these diseases can be understood on the level of the individual patient at the same moment that the patient is cured; many of these diseases seem to be clearly related to the repression of the individual character, as stressed already by Hippocrates. So if you simply start working with the patient to help him confront old existential pain and coach him in his personal development of his life by intensifying its meaning and purpose, the symptoms very often simply disappear. The toolbox of holistic medicine also seems relevant to even difficult, neurotic, psychosomatic, and hypochondriac patients. Believing in the treatment and not giving up on your patient, and moving forward in the treatment with the patient himself is the ultimate goal, even when you yourself do not understand the mechanism fully. This will force you to develop your own competence and is, in essence, what makes an outstanding holistic physician.

## INTRODUCTION

In the medical clinic, we are very often faced with health problems that do not have any known cause. The disease can be subjective, like pains that remain unexplained in spite of all examination programs; like tinnitus (noise in the ear) in a person who has not been exposed to noise, loud music, or anything else that can explain the condition; or the problem can be objective, like high blood pressure (hypertension) in a person who is otherwise healthy. Biomedicine is often unable to find a cure in these situations, leaving the problem unsolved or leaving the patient on a palliative pharmaceutical cure often lasting for lifetime. We believe that consciousness-based (holistic) medicine could be the choice for the patient and by using the theories and tool of holistic medicine[1,2,3] wisely, the physician could provide a cure for the patient. By helping the patient get rid of a symptom that you do not understand as a physician is intellectually upsetting, but the physician must give priority to the intention of healing the patient and helping him or her to avoid taking medication for life. It is quite a paradox that many of these diseases can be understood on the level of the individual patient in the same moment that the patient is cured. Many diseases seem to be related to the repression of the individual character, as stressed already by Hippocrates (460–377 BCE)[4]. Samuel Hahnemann (1755–1843)[5], the father of the controversial treatment system homeopathy, gave the following causes of diseases (first published in 1810), which still today are a fine supplement to our biomedical understanding and knowledge:

> It will help the physician to bring about a cure if he can find out the data of the most probable *occasion* of an acute disease, and the most significant factors in the entire history of a protracted wasting sickness, enabling him to find out its *fundamental cause*. The fundamental cause of a protracted wasting sickness mostly rests upon chronic miasm. In these investigations, the physician should take into account the patient's discernible body constitution (especially in cases of protracted disease) and
> 1. mental and emotional character [character of the *Geist* and the *Gemüt*],
> 2. occupations,
> 3. lifestyle and habits,
> 4. civic and domestic relationships [relationships outside and within the home],
> 5. age,
> 6. sexual function, etc.

Often, if you simply start working with the patient to help him confront old existential pain and coach him in his personal development of his life by intensifying its meaning and purpose, exploring the physical, mental, sexual, and spiritual character, the symptoms simply one day disappear. So do not give up on your patient, because one day your patient will probably be cured. Believing this and never giving up on the patient, moving forward in the treatment, even when you yourself do not understand fully and are forced to develop your own competence, is what makes an outstanding holistic physician.

## CLINICAL HOLISTIC MEDICINE

Interestingly, the toolbox of holistic medicine makes it possible to work with almost everybody because there is always something that can be improved if you look at the state of your patient: his love, his purpose of life and the way he uses his talents, his mind, his feelings, his body, and his sexuality. In an abstract sense, every patient will get the same treatment in the holistic clinic, meaning the healing of their fundamental existence in all the existential dimensions. In biomedicine, not understanding the disease and its etiology is a huge problem because you are then unable to find the right drug of choice. This does not matter as much for treatment in holistic medicine because you can always work with the patient on the intention of healing his or her life. To make this point clear, let us give a short review of our work in this field.

The life mission theory[6,7,8,9,10,11] states that everybody has a purpose of life or a huge talent. Happiness comes from living this purpose and succeeding in expressing the core talent in your life. To do this, it is important to develop as a person into what is known as the natural condition, a condition where the person know himself and uses all his efforts to achieve what is most important for him. The holistic process theory of healing[12,13,14,15] and the related quality of life theories[16,17,18] state that the return to the natural state of being is possible whenever the person gets the resources needed for existential healing. The resources needed are according to the theory of “holding” in the dimensions awareness, respect, care, acknowledgment, and acceptance with support and processing in the dimensions feeling, understanding, and letting go of negative attitudes and beliefs. The precondition for holistic healing to take place is trust and the intention that the healing will take place. Existential healing is not a local healing of any tissue, but a healing of the wholeness of the person, making him much more resourceful, loving, and knowledgeable of himself, his own needs and wishes. In letting go of negative attitudes and beliefs, the person returns to a more responsible existential position and an improved quality of life. The philosophical change of the person healing is often a change towards preferring difficult problems and challenges, instead of avoiding difficulties in life[19,20,21,22,23,24,25,26]. The person who becomes happier and more resourceful often also becomes more healthy, more talented, and more able to function[27,28,29].

Consciousness-based medicine is therefore about giving “holding” and processing to the patient and coaching him or her to lead a responsible and fine life with a high quality, talent, personal energy, and character. Improving quality of life seems to be the best medicine there is. We therefore often refer to our style of holistic medicine as “quality of life as medicine”[27,28,29].

## HYPERTENSION

Many people suffer from high blood pressure or hypertension. From the physiological point of view, this seems to involve overtensing of the smooth muscle cells of the arteries and, in particular, the very small arteries known as arterioles. No biochemical explanation has been found for this condition. A number of scientific studies have found strong evidence of a psychosomatic connection, as it has been possible for biofeedback to influence hypertension. The most feared consequence of hypertension is stroke, typically cerebral hemorrhages, which deprive the patient of mobility and sometimes proves fatal. A case of very high blood pressure is therefore always treated intensively by the family physician with drugs to lower blood pressure.

The holistic hypothesis for hypertension is that the small muscle cells in and around the blood vessels tense in order to hold onto gestalts in the same way as skeletal muscle can do so. Why gestalts can sometimes be brought about in the blood vessels is an unanswered question, but their location is presumably guided by the emotions involved. Experience indicates that many difficult emotions are associated with midlife crises, which settle in deep structures of the body such as the blood vessels and heart, while early fear of death from childhood settles low in the true pelvis.

It is easy in clinical practice to see the emotions that are linked to various tissues and organs as different, but typical emotional responses are obtained on contact with different areas of the body. There appears to be agreement among body therapists that the “internal waste bin” of the body becomes filled with traumas “from below”. In a corresponding way, breathing is blocked from below, so that a half-filled waste bin signifies that the patient only breathes down to the solar plexus. (EXERCISE: sit for 5 minutes and note your breathing. How far down into your body do you draw breath — to your neck, to your stomach, to your toes?)

The strategy adopted by the holistic physician in treating hypertension is to look for a repressed or hidden life crisis. Consciousness-based medicine does not need a hypothesis on localization, as all conditions involving suffering are always treated on the basis of the material supplied by the patient's own body and subconscious. Whatever emerges, the patient must feel, acknowledge, and let go.

We always combine consciousness-based medicine with biomedicine when we treat severe hypertension. When blood pressure is back to normal, the patient’s medication can be lowered or even discontinued. When we circle the patient’s relevant problems through the conversation, it is very clear when the problem is revealed. We can obtain a very direct perception of how this problem sits in the patient’s vessels and causes the hypertension. When the rise in blood pressure is more recent and comes in conjunction with particular changes in the patient’s life, it is possible to treat hypertension holistically.

Other times, no success is achieved in getting through with consciousness-based medicine. This happens particularly when blood pressure has been raised for years, without the patient himself having the slightest idea what has caused it. Hypertension, which often does not cause the patient any major bother, is difficult to treat holistically if the patient more or less unconsciously does not wish to think psychosomatically and see himself as the cause of the condition. Patients of this type will often be taking antihypertensive medication for the rest of their lives.

> **Male, aged 58 years, with hypertension:** First visit: Weight 97.2 kg. BP 210/110. Prescribe Centyl [bendroflumethiazide] + KCl and Atenor [atenolol] 50 mg. Another appointment to check BP. EXERCISE: Must draw up a plan to stop smoking and lose weight, “destressing” etc. Plan to retire at the age of 60 years?
>
> Second visit: Good progress. BP 150/90. The patient has reduced his consumption of cigarettes by half to ten a day since the last visit. He has had many useful thoughts about retirement and is getting a grip on it. We can discontinue his Atenor. Centyl + KCl. Weight 95.1 kg, which means that he has lost 1.8 kg. PLAN: Check again in 1 month, possibly reduce Centyl. Blood samples for electrolytes. The patient appears to be sad, but denies being so. Brief discussion about distance from emotions.

Here the patient discusses his existential problems with great passion and makes enormous progress after just one consultation. He has also made radical changes in his lifestyle and has started to lose weight. The reward comes immediately: His blood pressure normalizes, naturally also helped by biomedicine, which almost never, however, has as dramatic an effect on blood pressure as this. But, in the consultation he appeared sad even though he denied it. Often a patient of this kind will discover that he is terribly unhappy with a chronic feeling of unhappiness for many years. It may also be that he is unwilling to feel the sorrowful gestalt, which we as therapists can see in him.

It is also possible to project your own sorrow as a therapist onto him, which is the difficulty in this work. If this situation happens and we confuse our personal situation with that of the patient, it can cause serious problems and the physician or therapist may be obliged to offer a full apology for errors committed on this account. Fortunately, most patients forgive their therapist for committing errors, if they receive a sincerely meant apology.

> **Female, aged 46 years. Will blood pressure be reduced when her midlife crisis is treated?** Blood pressure measured three times 170/90 on both Centyl + KCl and Norvasc [amlodipine] 5 mg. The patient would like to know why she has high blood pressure. My hypothesis (SV) is that it may relate to a suppressed midlife crisis from 8 years ago, when she lost her job, which the body is still "holding onto". EXERCISE: Write down what was happening in that direction, when you originally had high blood pressure. Another appointment in 5 to 6 weeks.

It appears as though the traumas here are "laid down" in the vessel walls, which causes the muscle walls to tense chronically, so that the blood pressure rises unphysiologically. The acid test of the theory is obviously whether blood pressure drops again, when the crisis has been overcome. The difficulty is in making the patient open up to the old problems, which she has imagined to have been overcome long ago. That is not an easy task.

> **Man, aged 45 years, with hypertension:** BP today 170/115. Five minutes later 165/105. We add prescription of Centyl mite. Check again in 1 month. We talk a little about the cause of the hypertension and I (SV) suggest that he has some anxiety that he has been repressed. The patient disagrees.

You cannot win them all. No success was achieved here in involving the patient. It saddens us to think that he will probably be taking medication for many years to come and may perhaps have to put up with side effects from the medication, when he could have spent a few hours in his young life in coming to a personal understanding of why he has hypertension and doing something about it.

## TINNITUS

Tinnitus is the term used to describe an internal noise perceived by the patient, most often subjective. It can be very unpleasant with sometimes disabling, constant ringing in the ears. There is no effective biomedical treatment today apart from masking the tinnitus with noise supplied externally through a hearing aid. The disease is mysterious, since it may be accompanied by organic damage to the internal organs of the ear, but certainly need not be.

Patients often report that the activity of their tinnitus is variable and fluctuates according to their condition and "level of energy". Excessive strain or tiredness brings on the ringing. This ties in with the general experience that we need a surplus of mental energy to sense in a clear and well-defined way. When the personal level of energy falls, it is no longer possible to distinguish clearly with the senses and irrelevant signals emerge, because they are no longer automatically suppressed. Under normal circumstances, the brain suppresses all the noise that is not relevant, so that this noise does not reach our consciousness. We conjecture that it may be a suppression mechanism of this kind that fails in tinnitus.

Trying to understand tinnitus more precisely is detective work, even with a thorough knowledge of the structure of the ear and the function of the brain. The cochlea is the organ in the ear for sensing sound. It is built like a ladder, so that rapid vibrations — which strike the inner ear from the tympanic membrane via the malleus, incus, and stapes — migrate furthest owing to the smaller wavelength. The vibrations then cause hairs known as cilia on the sensory cells to vibrate and the cell fires off nerve signals, which reach the brain through several relay nuclei and are converted into sound. But why does this ringing occur in the brain in patients with tinnitus when the fibers are *not* vibrating? If we look at nerve fibers, which pass between the cochlea and the brain, it appears that the brain has very great freedom to modify the signal coming from the ear. The cochlea, which apparently supplies the sound in tinnitus and therefore appears to be the source of the problem, has ten times as many *afferent* nerve fibers as *efferent* ones, and a hypothesis for tinnitus is therefore that it is a faulty signal from the brain that causes the nerve cells in the auditory ganglia to "ring". This mechanistic track, as is so often the case, close in a blind alley or dead end: the cause of tinnitus remains unknown.

While the mechanistic explanatory model of biomedicine has to concede defeat, holistic medicine can provide the theory for tinnitus as a blockage in the relevant tissue in the ear or brain that gives rise to the symptoms. This explanation is not satisfactory, but is sufficient for it to be possible for holistic treatment to be implemented in practice. Blockages are due to painful emotions, which have been parked in the tissue, so that the patient cannot feel them. When they are cancelled out, the function of the cells and tissues, which are not working properly, is re-established and the tinnitus disappears. This fits in with our clinical experience. Interestingly enough, a great deal of progress is often made by the simple “laying-on of hands” — a classical medical approach — over the ear cartilage and the surrounding cranial bones, but such a maneuver rarely has a lasting effect. Our experience is that instead of working locally around the ear, it is a good idea to implement holistic treatment from the outset. It is advisable always to identify and process the repressed incidents and emotional pain, regardless of where in the body and mind these blockages present themselves. When the internal waste bin has been sufficiently emptied, the symptoms of tinnitus according to this theory will be alleviated; this applies almost regardless of what symptom is concerned.

> **Female, aged 25 years, from pain in the ear to personal development:** First visit: 1. Pain in the ear for 24 days and tinnitus in right ear. Otoscopy shows unilateral red tympanic membrane /Otitis media dex/ /tinnitus dex/ Prescribe Penicillin. 2. Personal problems, which she would like to talk about. Appointment for conversation.
>
> Second visit: She has lots of problems and is totally unable to gain an overview of them. Did not benefit from going to see a psychiatrist and does not know where to turn next. She is very anxious to avoid conflict, feels dominated by her parents, who interfere in everything. She feels very poorly, but cannot put into words what is wrong or about her bad emotions. Feels lonely and different, would like to have a boyfriend. Feels as though she is inside the dome of a cheese dish, with no contact. Is not at peace with herself. Feels that she thinks differently than many other people and has different interests. Feels an emptiness, which she cannot explain. Feels that she has loads of self-confidence and can do lots of things, but is completely incapable of action. EXERCISE 1 – thought: Make a complete list of all your problems – and write down the history of these problems by next time. EXERCISE 2 – emotion: When things go badly, sit down and feel what kind of emotions it is that are bothering you. Get to know them rather than escaping from them.
>
> Third visit: 1. Smear. Pelvic examination: Inspection: vulva, vagina, vaginal orifice natural. Exploration: No cervical motion tenderness corresponding to the uterus, no tenderness from adnexa. 5/30 days, last menstrual period 14 days ago. 2. Hearing problems. Otoscopy shows lesion of one of the eardrums with “discharge from ear”. Prescribe Penicillin. 3. Conversation on the patient’s problems, which appear to be that she suppresses her own wishes and needs. Asthma treatment: she is a heavy user of Bricanyl [terbulatine] at present, several times daily. Has completely stopped Spirocort [budesonide]. Prescribe Symbicort [budesonide + formoterol] 2 puffs daily for 2 weeks, then one puff twice daily. She should attend for spirometry in the near future.
>
> Fourth visit: Conversation about personal development. The patient feels empty and would like to do something about it. We talk about how she can proceed.

The patient presents with earache, ear infection, and tinnitus. She accepts an existentially oriented conversation. It is found that she has an inconceivable number of problems and was sent home to work towards getting a grip on them herself. She did that and acknowledged that she suppresses her own needs. Underneath there is a more fundamental lack of meaning in life, which is experienced as emptiness. She is now involved, assisted by her new consciousness, in an actual process of personal development. The physical problems have completely disappeared from focus. The tinnitus would probably also have disappeared without psychosocial intervention, but not until later.

> **Male, aged 58 years, creeping decline with tinnitus:** 1. Tired the whole time, worst in the morning – has gradually worsened over the last year since early retirement. We order blood tests to rule out serious disease; if the patient is not somatically ill, depression must be considered. 2. Shoulder pain in the form of tension. Can take an extra Nobligan [tramodol] when needed. 3. He has a howling noise in his ear. He finds it difficult to talk to other people because of the noise, whistling, and howling. /Tinnitus/Order audiometry to assess need for hearing aid, if necessary with masking for tinnitus.

## DISCUSSION

It is so distressing to see people decline. There is nothing necessary about this continued decline and there is no biological necessity, but people have to mobilize their internal willpower to return to life themselves. It cannot be supplied from the outside. When people make themselves weak by accepting and accepting and accepting without rebellion or fight and lack of initiative, there is not much that we as physicians can do. People decide for themselves whether they want to live or die. There is no force in the world that can alter that. We are created autonomous; we decide for ourselves.

Tinnitus seems to appear as a symptom of blockages related to the ear combined with general weakening. From our experience, it seems that patients who work with themselves become free of their tinnitus, while those who cannot be motivated to work on their old pains and fundamental gestalts retain their tinnitus. Very often in the medical clinic, we face health problems of our patients that do not have any known cause. Biomedicine is often unable to find a cure in these situations, leaving the problem unsolved or leaving the patient on a palliative pharmaceutical cure often for a lifetime. In this case, consciousness-based (holistic) medicine is often the patient's choice and using the theories[6,7,8,9,10, 11,14,15] and tools of holistic medicine[1,2,3] wisely, the physician can provide a cure for the patient in many cases. Helping the patient to get rid of a symptom that you do not understand as a physician is intellectually upsetting, but the physician must give priority to the intention of healing the whole patient.

The toolbox of holistic medicine makes it possible to work on almost everybody because there is always something that can be improved if you look at the state of your patient: his love, his purpose of life, and the way he uses his talents, his mind, his feelings, his body, and his sexuality. In an abstract sense, every patient in the world will get the same treatment in the holistic clinic: healing their fundamental existence in all existential dimensions. In biomedicine, not understanding the disease or its etiology creates a huge problem because you cannot pick the right drug to use. In holistic medicine, this does not really matter for the treatment because you can always work on the patient with the intention of healing his or her life.

It is quite a paradox that many of these diseases can be understood on the level of the individual patient in the same moment that the patient is cured. Many diseases seem to be clearly related to the repression of the individual character, as stressed already by Hippocrates. Very often, if you simply start working with the patient to help him confront old existential pains and coach him in his personal development of his life, intensifying meaning and purpose, the symptoms simply one day disappear.

So using the toolbox of holistic medicine, you do not have to give up on your difficult patient because you can assist the patient. Believe this and never give up on the patient, always move forward in the treatment with the patient himself as the ultimate goal, even when you yourself do not understand fully the mechanism. This way, you are forced to develop your own competence, which makes an outstanding physician.

So giving "holding", care, and processing to our patient and coaching him or her to lead a responsible and fine life with high quality, using his talents, personal energy, and character is what consciousness-based medicine is all about. Improving quality of life seems to be the best medicine. We therefore often refer to our style of holistic medicine as "quality of life as medicine"[27,28,29].

## ACKNOWLEDGMENTS

This study was supported by grants from IMK Almene Fond. The quality of life research was approved by the Copenhagen Scientific Ethical Committee under number (KF)V.100.2123/91.

## BIOSKETCHES

**Søren Ventegodt, MD,** is the Director of the Quality of Life Research Center in Copenhagen, Denmark. He is also responsible for a Research Clinic for Holistic Medicine in Copenhagen and is a popular speaker throughout Scandinavia. He has published numerous scientific or popular articles and a number of books on holistic medicine, quality of life, and quality of working life. His most important scientific contributions are the comprehensive SEQOL questionnaire, the very short QoL5 questionnaire, the integrated QOL theory, the holistic process theory of healing, the life mission theory, and the Danish Quality of Life Research Survey, 1991–94 in cooperation with the University Hospital of Copenhagen and the late pediatric professor Bengt Zachau-Christiansen. E-mail: ventegodt@livskvalitet.org. Website: http://www.livskvalitet.org

**Mohammed Morad, MD**, is Specialist in Family Medicine, Lecturer in Family Medicine at the National Institute of Child Health and Human Development, Division of Community Health, Ben Gurion University of the Negev and the Medical Director of a large area clinic in the city of Beer-Sheva. He has publications on Bedouin health, health aspects, spiritual health, and aging in persons with intellectual disability, and is a presenter on topics such as health policy and services for the disadvantaged at national and international conferences. E-mail: morad62@barak-online.net

**Isack Kandel**, **MA, PhD**, is Senior Lecturer at the Faculty of Social Sciences, Department of Behavioral Sciences, the Academic College of Judea and Samaria, Ariel. During the period 1985–1993, he served as the Director of the Division for Mental Retardation, Ministry of Social Affairs, Jerusalem, Israel. E-mail: Kandeli@aquanet.co.il

**Joav Merrick, MD, DMSc,** is Professor of Child Health and Human Development affiliated with the Zusman Child Development Center, Division of Pediatrics and Community Health at the Ben Gurion University, Beer-Sheva, Israel; the Medical Director of the Division for Mental Retardation, Ministry of Social Affairs, Jerusalem; and the Founder and Director of the National Institute of Child Health and Human Development. He has numerous publications in the field of child and human development, rehabilitation, intellectual disability, disability, health, welfare, abuse, advocacy, quality of life, and prevention. Dr. Merrick received the Peter Sabroe Child Award for outstanding work on behalf of Danish Children in 1985 and the International LEGO-Prize ("The Children's Nobel Prize") for an extraordinary contribution towards improvement in child welfare and well being in 1987. E-mail: jmerrick@internet-zahav.net. Website: www.nichd-israel.com
